# A Controlled Single-Speaker Evaluation of Ambient Voice Technologies’ Speech-to-Text Function Using Narrated Surgical Case-Reports

**DOI:** 10.1007/s10916-026-02471-5

**Published:** 2026-10-09

**Authors:** Ruairi O’Kane, Daniel Stonehouse-Smith, Melody Shirazi, Rachel Hutchinson, Morgan Gregg, Riya Patel, Jadbinder Seehra, Spyridon N Papageorgiou, Martyn T Cobourne

**Affiliations:** 1 Faculty of Dentistry, Oral & Craniofacial Sciences, Centre for Craniofacial Development & Regeneration, King’s College London, Floor 27, SE1 9RT London, UK; 2 Department of Orthodontics, Guy’s & St Thomas’ NHS Foundation Trust, London, UK; 3 Department of Oral & Maxillofacial Surgery, King’s College Hospital NHS Foundation Trust, London, UK; 4 Department of Orthodontics, Royal Alexandra Children’s Hospital, Sussex University Hospitals NHS Foundation Trust, Brighton, UK; 5 Centre of Dental Medicine, Clinic of Orthodontics and Paediatric Dentistry, University of Zurich, Zurich, Switzerland

**Keywords:** Speech recognition software, Surgery, Artificial intelligence, Natural language processing, Medical records, Transcription accuracy

## Abstract

Ambient voice technologies (AVT) utilise automatic speech recognition (ASR) and generative artificial intelligence for speech-to-text conversion, and are advocated as efficient, accurate tools for clinical documentation. We investigated (*n* = 8) AVT systems using surgical case-reports as narratives in a controlled single-speaker evaluation prioritising safety outcomes: specifically 3 commercial transcription-systems (Dragon-Medical-One, Heidi-Health, Tortus); 4 ASR speech-to-text Application Programming Interfaces (Speechmatics-Enhanced, Amazon-Medical-Transcribe, Whisper, GPT4oTranscribe); and an experimental two-stage ASR–Large Language Model (LLM) pipeline incorporating GPT4oTranscribe with GPT-5-LLM generative error correction (GPT4oTranscribe-Corrected-5). Reference-transcripts (*n* = 100; 32,897 words, range = 44–449, mean = 329/per-transcript) derived from surgical case-reports were recorded and input into AVT systems as audio-recordings for transcription-output generation. Primary outcome was proportion of transcription-outputs containing at least one clinically significant Class 3 error graded for potential harm. Secondary outcomes included transcription accuracy: Domain-Word-Error-Rate (DWER) against SNOMED-CT, lexical-accuracy (ROUGE score) and semantic similarity (BERT, BART scores). To investigate impact of the experimental pipeline on clinically significant errors, raw ASR and LLM-processed transcription-outputs were compared and errors classified as resolved, remaining or newly introduced. Across (*n* = 800) transcript-outputs, 30–68% (GPT4oTranscribe-Corrected-5; Amazon-Medical-Transcribe and Dragon-Medical-One, respectively) contained at least one clinically significant Class 3 error. LLM-processing reduced the proportion of transcript-outputs affected (53% to 30%), amongst 89 clinically significant errors in ASR-output, 51 were resolved, 38 remained significant and 4 were newly introduced. Reference-transcript length significantly increased odds of Class 3 errors for 4 systems (GPT4oTranscribe, Tortus, Amazon-Medical-Transcribe, Dragon-Medical-One; odds ratio 2.65–3.34/additional 100 reference-words). Errors with potential to cause severe harm or death (NHS England Level 3–4) totalled (*n* = 53) across systems, concentrated in the domains of medication-type/dose (39.6%), and investigations/laboratory results (30.2%). Performance varied across systems for transcription metrics (*P* < 0.001). GPT4oTranscribe-Corrected-5 (DWER = 3.60%) and Heidi-Health (DWER = 5.67%) performed best for medical terminology; Amazon-Medical-Transcribe worst (DWER = 24.03%). For lexical and semantic similarity GPT4oTranscribe-Corrected-5 performed best, followed by Heidi-Health. The ASR–LLM pipeline reduced clinically significant errors but did introduce new ones in 3% of transcript-outputs. The safety and effectiveness of these systems in clinical practice requires further evaluation.

**Supplementary Information:**

The online version contains supplementary material available at https://doi.org/10.1007/s10916-026-02471-5.

## Introduction

Ambient voice technologies (AVT) represent documentation tools that use generative artificial intelligence (AI) to capture and transcribe dictated and conversational speech. These applications are increasingly advocated for use in healthcare to scribe clinical notes, reducing clinician time spent on documentation and potentially improving efficiency [1–5]. Their uptake reflects a broader issue of administrative burden for healthcare providers [6], problems of clinician-burnout, reduced standards of care and patient-safety issues [7, 8] but commercial availability and deployment has outpaced the available evidence on accuracy and safety.

In the United Kingdom, the Royal College of Surgeons of England *Good Surgical Practice* sets out expectations for accurate, comprehensive and contemporaneous record-keeping in the surgical specialties [9]. National Health Service (NHS) England guidance on AI-enabled ambient-scribing for Electronic Healthcare Records (EHR) outlines clear expectations for deployment in clinical practice [10]. Specifically, outputs should be reviewed by a clinician before they are embedded within the care pathway; particularly for transcription accuracy and identification of errors (such as missing or incorrect information). Recent systematic review of AVT in EHR has highlighted an imbalance between implementation efficiency outcomes and evidence on accuracy across clinical settings [11, 12]. Indeed, reported error rates can vary widely, with stronger performance in dictation but poorer outcomes for conversational, multi-speaker or specialty-specific speech, which frequently require substantial post-transcription editing [12]. Implementation studies consistently describe reduced documentation-time and improved clinician experience as key advantages of these systems but provide little robust data on the fundamental requirements of transcription accuracy and absence of clinically relevant errors, and evidence in surgical domains is particularly sparse [11].

Automatic speech recognition (ASR) is the process by which spoken language is converted into transcribed text and forms the speech-to-text component of AVT. Many contemporary systems are made available through speech-to-text Application Programming Interfaces (API). These newer tools should be more adept at understanding context and specialised terminology, and recent research has demonstrated their efficiency in medicine [13, 14]. However, ASR performance can be influenced by multiple variables, including accent, background noise and technical language (particularly clinical terminology), and collectively, these limitations can shape the type of errors that appear in clinical notes generated by AVT [15, 16]. Moreover, the practice of surgery provides a unique set of challenges for AVT, representing a subject with highly specialised technical language deeply embedded within clinical anatomical nomenclature, spanning multiple individual specialties. Errors in operative clinical notes can have significant consequences, affecting diagnosis, treatment, post-operative follow-up, and potentially misleading subsequent clinicians.

Whilst transcription accuracy metrics provide a useful summary of fidelity, they can fail to capture the clinical significance of mistranscriptions. A recent scoping review found that most systems remain at early technology-readiness levels with patient-safety outcomes largely absent [17]. Indeed, recent work within dentistry using orthodontic clinical records has shown that even when word error rates are low, AI-generated transcription can still introduce significant errors, capable of altering the accuracy, interpretation and overall quality of EHR [16]. Concerns about the clinical impact of transcription errors are supported by evaluations of earlier speech recognition systems, including analyses of medicolegal claims where documentation errors contributed to patient harm [18]. The correction of ASR output has been evaluated previously using fine-tuned sequence-to-sequence models and prompted Large Language Models (LLM) [19, 20] and GPT-4o correction on raw ASR outputs can be beneficial in the context of orthodontic records [16]. More recent work has evaluated the effect of LLM post-processing in improving accent-related accuracy for non-native English speakers [21]. However, in general the clinical impact of LLM processing in medical transcription is poorly characterised.

Here, we evaluated eight contemporary AVT for dictation of surgical clinical records, using the proportion of transcription-outputs containing at least one clinically significant error as the primary outcome, graded for potential harm to determine whether current outputs are compatible with existing standards for safe documentation and deployment. We also included a two-stage ASR–LLM experimental pipeline and additionally performed paired error-level analysis to determine if clinically significant errors were resolved, if they remained or if any new errors were introduced. We also report on transcriptional, lexical and semantic accuracy of dictated transcripts, and investigated the influence of dictation length (which is a property of documentation rather than AVT system) on per-recording risk of error, which has relevance for post-deployment monitoring. This investigation is restricted to the speech-to-text component of AVT, assessing accuracy of clinician-transcribed notes rather than summarisation.

## Materials and Methods

### Study Design and Ethics

We evaluated AVT speech-to-text function using surgical case-reports as narratives read by a single speaker. This study followed the MINimum Information for Medical AI Reporting (MINIMAR) reporting standards for AI in healthcare [22]. Ethical approval was granted by King’s College London as a minimal-risk investigation (MRA-24/25-53054). A detailed description of the materials and methods is available in the supplementary material.

### Dataset Creation and Sample Size

A corpus of 100 written case-reports from open access surgical journals indexed in PubMed Central was used for transcription, with each describing clinical presentation, operative management and early postoperative course. These case-reports served as the written ground truth reference-transcripts for all analyses and consisted of (*n* = 32,897) words in total, range (*n* = 44–449), mean (*n* = 329) words per case-report. Each reference-transcript was narrated by a single speaker (RO) in a controlled environment at King’s College London to produce (*n* = 100) corresponding WAV audio-recordings. These audio-recordings were then input into the speech-to-text function of the AVT systems being tested and the subsequent transcript-outputs investigated.

In order to validate accuracy of audio-recordings (to ascertain that errors in the transcription-output reflected AVT system transcription errors rather than dictation errors in the audio-recording), two external reviewers (MG and RP) manually and independently compared a random sample of (*n* = 20) audio-recordings with the corresponding reference-transcripts and collated any differences. A total of (*n* = 2) word errors were identified across (*n* = 5,932) written words in the sample (0.034% error rate). This was below the 0.5% maximum transcription error rate proposed for validating speech transcripts [23]. These identified errors were corrected in the relevant reference-transcripts. Full search, selection and recording methods are given in the supplementary material.

The initial sample size was calculated based upon data from our previous study [16] using a chi-square test for equality of proportions of clinically significant Class 3 errors across the eight groups, as described by Cohen for effect size *w* in contingency [24]. To account for clustering, the required sample size was inflated using the standard design effect formula [25]. We calculated an intraclass correlation coefficient of 0.30 estimated from previous data investigating AVT systems in clinical dentistry [16] using a mixed-effects logistic regression model with a random intercept for recording, which was inflated to 0.45 to account for potential field-specific differences. The final sample size was obtained by multiplying the independent-sample estimate by the design-effect. All calculations assumed a two-sided α = 0.001 and 99% power and resulted in a requirement for (*n* = 72) recordings, which was rounded up to (*n* = 100) to also account for the effect of word number per reference-transcript text length on Class 3 error.

### Ambient Voice Technology Systems

We evaluated eight contemporary AVT-systems with selection based upon accessibility, functionality and relevance to clinical practice. We included (*n* = 3) commercial systems: Nuance Dragon Medical One (Microsoft Corp) (a dictation product trained for healthcare terminology), Heidi-Health (Heidi-Health) and Tortus (Tortus AI) (ambient digital-scribes designed for clinical use); and (*n* = 4) standalone ASR systems accessed through speech-to-text APIs: Speechmatics Enhanced (Speechmatics), Amazon Medical Transcribe (Amazon), Whisper (OpenAI), GPT4oTranscribe (OpenAI). We also evaluated an experimental two-stage ASR–Large Language Model (LLM) pipeline, in which the GPT4oTranscribe transcription-output underwent generative error correction (GEC) using a LLM (GPT-5) and previously evaluated prompt (GPT4oTranscribe-Corrected-5). The raw GPT4oTranscribe output was given to the LLM as text through the OpenAI Chat Completions endpoint (/v1/chat/completions). The full prompt and model configuration data are provided in the supplementary material [see also 16] (Supplementary Figure S1). The (*n* = 800) transcription-outputs derived from the eight AVT investigated were then compared with the original written reference-transcripts.

### Primary Outcome

Primary outcome of this investigation was the proportion of transcription-outputs containing at least one clinically significant Class 3 error. A Class 3 error, under the modified Kanal error analysis [26] is a transcription error changing meaning in a way that is not obvious on immediate inspection, and defined as clinically significant if it could lead a reader to misunderstand the clinical meaning and potentially impact patient care [27]. Two reviewers manually annotated errors and assessed clinical significance of each Class 3 error. Raw agreement between reviewers for the binary primary outcome (Class 3 clinically significant error versus all other classifications) was 96.61% with Cohen’s Kappa κ = 0.923 and across the five-category Kanal Class (Class 0–2, Class 3 not clinically significant, Class 3 clinically significant) was 95.69% with Cohen’s Kappa κ = 0.806, indicating substantial agreement [28] (Supplementary Table S1). Any queries or discrepancies were resolved by a third reviewer. Clinically significant Class 3 errors were then categorised by error domain and potential harm-severity. To assess severity (considering the plausible consequence if the error was propagated uncorrected and acted upon) a four-level scale aligned with the NHS England Harm Framework [29] was used. The degree of potential harm was recorded as Level 1 (low), Level 2 (moderate), Level 3 (severe/serious deterioration) or Level 4 (death). For the paired comparison of raw GPT4oTranscribe ASR output and the LLM-processed experimental pipeline output (GPT4oTranscribe-Corrected-5), each clinically significant error was classified according to its outcome after LLM-processing as resolved, remaining clinically significant or newly introduced.

### Secondary Outcomes

**Fig. 1:**
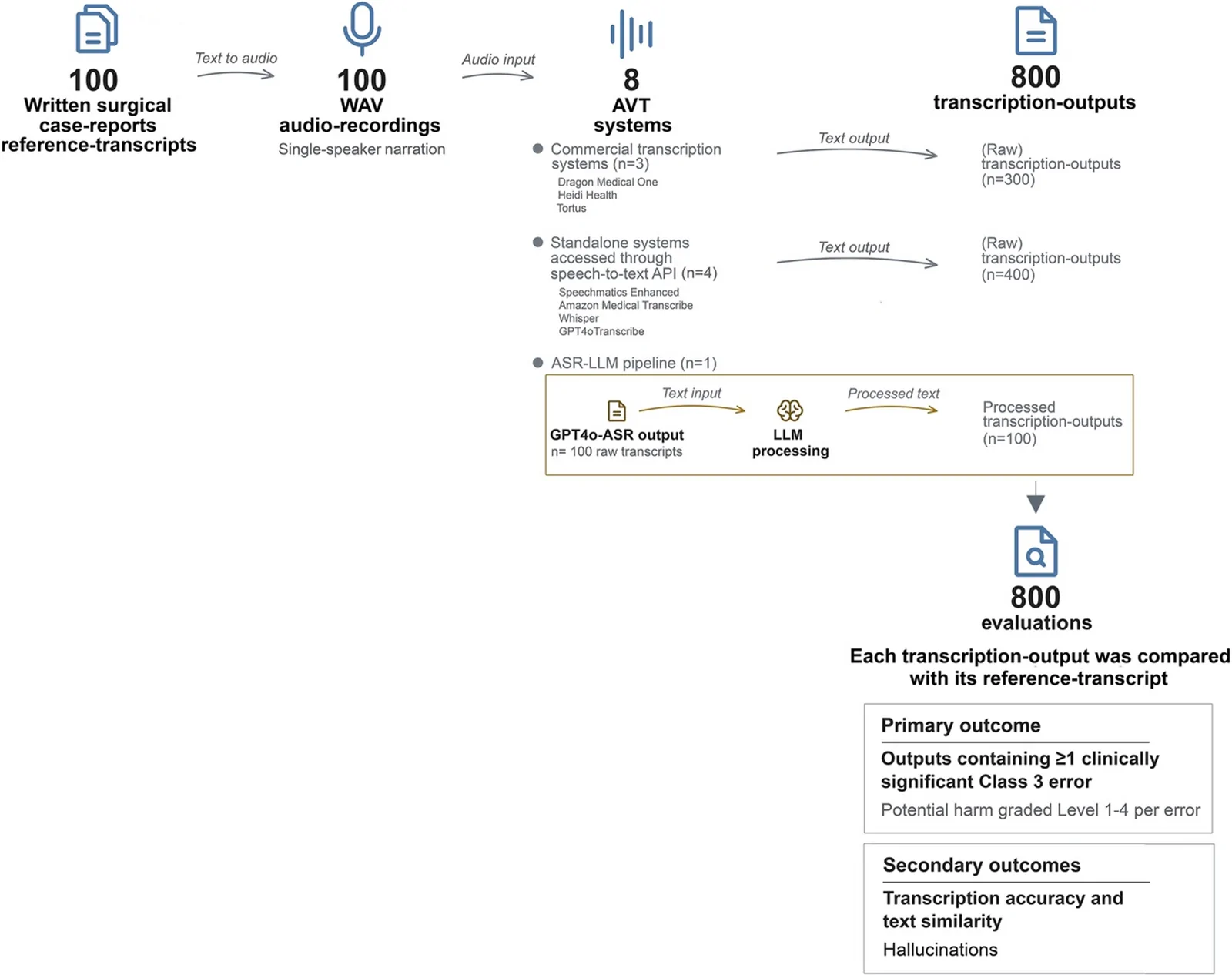
Schematic demonstrating the study workflow. One hundred written surgical case-reports representing the reference-transcripts were recorded as WAV files by a single speaker with these audio-recordings processed by eight ambient voice technologies (AVT) speech-to-text systems. These included 3 commercial transcription systems, 4 standalone systems accessed by speech-to-text application programming interface (API) and 1 experimental pipeline combining automated speech recognition (ASR) and Large Language Model (LLM) processing. Each output (both raw and processed) was compared with its reference-transcript to assess clinically significant Class 3 errors and their potential harm (primary outcome), and overall transcription accuracy and text similarity (secondary outcomes)

Word Error Rate (WER) is a widely employed metric in ASR that quantifies overall transcription accuracy. Although WER provides an overall measure of performance, it may insufficiently capture domain-specific nuances and does not consider the importance of individual words or the potential impact of errors [30]. We therefore calculated Domain Word Error Rate (DWER) because this metric extends WER by focusing on errors associated with clinical terminology, and accuracy for these terms is critical for a medical scribe. DWER was calculated by focusing on clinical terminology vocabulary derived from Systematized Nomenclature of Medicine Clinical Terms (SNOMED-CT) International Edition, English Description snapshot (release 2025-08-01), a clinical vocabulary used in EHR [31]. Domain Term Missed Ratio alternatively, focuses on the number of occasions in which a particular clinical term was transcribed incorrectly. Additional secondary outcomes included Non-Domain WER (N-DWER) which quantifies errors in general language excluding clinical terms, unnormalised WER (uWER) which penalises formatting errors and Character Error Rate (CER) for per-character transcription errors. For lexical similarity, ROUGE (Recall-Orientated Understudy for Gisting Evaluation) score [32] was used, as well as BERT (Bidirectional Encoder Representations from Transformers) and BART (Bidirectional and Auto-Regressive Transformer) scores for measurements of semantic similarity [33, 34]. Error analysis also included manual review of Hallucinations (transcribed content absent from the dictated audio). The evaluation metrics used in this investigation are summarised in Table 1 and the study workflow is shown in Fig. 1.

**Table 1:** Summary of evaluation metrics and their interpretation

Metric	Definition and interpretation
System outputs containing at least one clinically significant error, n (%)	Number and percentage of transcription-outputs containing at least one non-obvious error that changes clinical meaning in a way to potentially impact patient care
Potential Harm Level, 1–4	The potential consequence which could plausibly occur if a clinically significant mistranscription was left uncorrected
Word Error Rate (WER), %	The number of words either transcribed incorrectly, added or omitted as a percentage of reference words after normalisation
Domain Word Error Rate (DWER), %	Errors involving SNOMED-CT derived clinical words which were transcribed incorrectly, added or omitted
Non-Domain Word Error Rate (N-DWER), %	The number of general language words transcribed incorrectly, added or omitted
Unnormalised Word Error Rate (uWER), %	The same metric as WER before standardising capitalisation, punctuation, numbers and units
Character Error Rate (CER), %	The number of characters either transcribed incorrectly, added or omitted as a percentage of the total number of characters in the reference
Domain Term Missed Ratio, %	How often clinical terms were transcribed incorrectly
ROUGE	ROUGE (Recall-Oriented Understudy for Gisting Evaluation) Word or phrase overlap with the reference text with higher scores indicating closer lexical similarity
BERT Score	BERT (Bidirectional Encoder Representations from Transformers) Semantic similarity between the reference text and the transcript-output with higher scores indicating closer meaning
BART Score	BART (Bidirectional and Auto-Regressive Transformer) Likelihood of the reference given the transcription-output, with higher (less negative) scores indicating greater semantic similarity
Outputs containing at least one hallucination, n (%)	The introduction of incorrect or misleading information that was not present in the spoken audio

### Statistical Analysis

After testing for normality with the Shapiro-Wilk test, descriptive statistics were calculated included medians with Interquartile Ranges (IQR) or absolute/relative frequencies. Differences between DWER and N-DWER for each tool were investigated using the Wilcoxon-signed test, followed by effect size estimation. Differences across tools in proportion of transcripts with clinically significant Class 3 errors and in performance metrics (WER, DWER, N-DWER, uWER, CER, average ROUGE, ROUGE-1, ROUGE-2, BERT, and BART) were assessed with Generalized Linear Models (GLM) with a binomial distribution and a logit link function or median conditional quantile regression (Machado–Parente–Santos Silva robust variance estimator), with cluster-robust variance estimation (Huber–White sandwich estimators) clustered at the reference recording level, and expressed as unstandardised regression coefficients or odds ratios (OR) with their 95% Confidence Intervals (CI). After overall differences across all tools with Wald-type tests, pairwise comparisons to the best-performing tool were done with Bonferroni correction (by keeping alpha set and adjusting the p-value). The proportion of transcripts containing at least one clinically significant Class 3 error was calculated for each AVT-system again adjusted for within-recording clustering via robust standard errors. The association between length of the surgical record and the presence of at least one clinically significant error was assessed per additional 100 words with GLM. Inter-rater reliability between the two reviewers across all transcripts was evaluated using Cohen’s kappa. For the binary variable, unweighted kappa was reported, while for the Kanal Class variable, weighted kappa with quadratic weights was applied, while for clinically significant errors an unweighted kappa was reported. 95% CIs were estimated using bias-corrected non-parametric bootstrapping with 2,000 replications. All analyses were run in Stata SE 19.5 (StataCorp, College Station, TX, USA) with a two-sided alpha set at 5% and an open dataset depsited at: https://doi.org/10.5281/zenodo.18209141.

## Results

Across the (*n* = 800) transcription-outputs generated from 100 audio-recordings across the (*n* = 8) AVT systems, the proportion containing at least one Class 3 clinically significant error varied substantially, ranging from 30% (GPT4oTranscribe-Corrected-5) to 68% (Amazon Medical Transcribe, Dragon Medical One) (Table 2). Length of narration was significantly associated with differential AVT performance, as seen by a significant interaction term. Stratification according to AVT system indicated that the performance of some was not associated with narration length (GPT4o Transcribe-Corrected-5, Whisper, Speechmatics-Enhanced, Heidi-Health; all *P* ≥ 0.05). However, for four AVT systems, each additional 100 dictated words significantly increased the odds of a clinically significant Class 3 error: GPT4oTranscribe (OR 2.65; 95% CI 1.32 to 5.32), Tortus (OR 3.34; 95% CI 1.61 to 6.90), Amazon Medical Transcribe (OR 2.85; 95% CI 1.45 to 5.60), and Dragon Medical One (OR 2.98; 95% CI 1.55 to 5.71) (*P* < 0.05 for all) (Supplementary Table S2).

**Table 2:** Summary of Class 3 clinically significant errors across AVT systems

	Total errors (*n*)	Number of Class 3 clinically significant errors (*n*)	Transcript-outputs with at least 1 clinically significant error, % [95% CI]	OR (95% CI)	*P* value
Amazon Medical Transcribe	4,147	145	68.0 [58.1–76.5]	5.45 (3.06–9.71)	< 0.001^a^
Dragon Medical One	3,818	177	68.0 [58.1–76.5]	5.45 (3.06–9.69)	< 0.001^a^
GPT4o Transcribe	2,811	89	53.0 [43.1–62.7]	2.78 (1.78–4.33)	< 0.001^a^
GPT4oTranscribeCorrected-5	1,248	42	30.0 [21.7–39.8]	Reference	-
Heidi-Health	1,528	89	54.0 [44.0-63.7]	2.90 (1.75–4.81)	< 0.001^a^
Speechmatics enhanced	2,850	87	48.0 [38.2–57.9]	2.25 (1.29–3.90)	0.02^a^
Tortus	3,417	143	62.0 [52.0-71.1]	4.11 (2.39–7.08)	< 0.001^a^
Whisper	3,337	77	44.0 [34.5–54.0]	1.89 (1.13–3.17)	0.11^a^

Total Errors are the sum of Class 0–3 errors for each system. *n* = 100 transcripts per ASR system

*CI *confidence interval, *OR *odds ratio

^a^ Bonferroni correction applied

Overall system effect (adjusting for word count): Wald χ²(7) = 51.79, *P* < 0.001

In the experimental (GPT4oTranscribe-Corrected-5) pipeline, GPT-5 LLM-processing of the raw GPT4oTranscribe ASR transcript-output reduced the proportion of outputs containing at least one clinically significant Class 3 error from 53% to 30% (OR 0.38, 95% CI 0.25–0.58; *P* < 0.001) (Supplementary Table S3). Of the (*n* = 89) clinically significant errors in the raw ASR output, LLM-processing resolved (*n* = 51; 57.3%) of these and (*n* = 38; 42.7%) remained clinically significant. However, there were four clinically significant errors introduced by LLM-processing (Supplementary Table S4). Clinically significant errors inconsistent with the clinical context of the case-report were resolved, such as changing the incorrect ‘flucocytosine’ to the correct ‘flucloxacillin’ in regard to the treatment regimen for methicillin-sensitive *Staphylococcus aureus.* Nonetheless, numerical errors in ASR transcription-output would often persist such as, ‘6%’ remaining incorrect after processing against the reference-transcript, ‘60%’. Despite a reduction in clinically significant errors, there were unsupported changes which introduced new examples, such as replacing ‘malformation’ with ‘malignant’ (Supplementary Table S4).

**Fig. 2:**
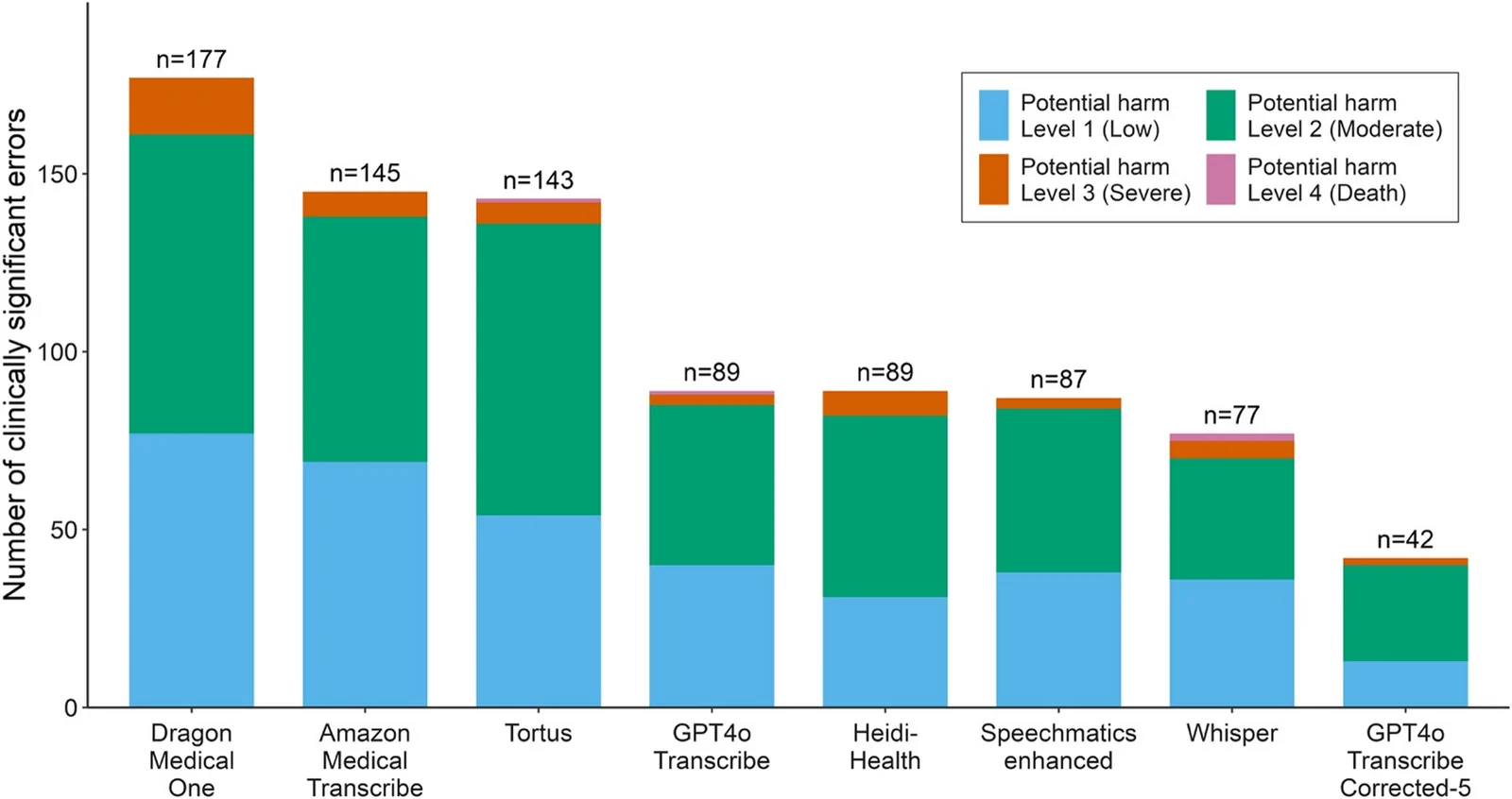
Potential harm severity distribution by AVT for clinically significant Class 3 errors. Bars show the number of clinically significant Class 3 errors per AVT stratified by potential harm level (Levels 1–4)

Total number of errors are shown in Table 2 and ranged from (*n* = 1,248) to (*n* = 4,147) (GPT4o-Transcribe-Corrected-5; Amazon Medical Transcribe, respectively). The number of clinically significant errors per system ranged from (*n* = 42) to (*n* = 177) (GPT4o-Transcribe-Corrected-5; Dragon Medical One, respectively). Examples of Class 3 clinically significant errors across domain categories are shown in Table 3 with the distribution of errors by potential harm severity shown in Fig. 2 (and Supplementary Table S5). Clinically significant errors were most frequently observed in association with clinical investigations and laboratory results (30.4%), patient history and background (17.9%), medication type and dose (13.2%), and procedure and operative detail (16.1%). Severe errors (potential harm Level 3 and 4) were uncommon overall, with a total (*n* = 53) across AVT. Errors involving medication-type and/or dose (*n* = 21, 39.6%), investigations and laboratory results (*n* = 16, 30.2%) were most frequent (Figure S2).

**Table 3:** Examples of Class 3 clinically significant errors across domain categories

Domain category	Example		% of Class 3 clinically significant errors
	Reference-transcript	Transcript-output	
Patient history and background	*‘65-year-old woman with chronic obstructive pulmonary disease fibromyalgia* *hypothyroidism* *and oesophageal dysmotility presented with 2 weeks of left sided chest pain’*	*‘65-year-old woman with chronic obstructive pulmonary disease fibromyalgia*, *hyperthyroidism*, *and oesophageal dysmotility presented with 2 weeks of left sided chest pain’.* Heidi Health, Transcript 9	17.9
Investigations and laboratory results	*‘creatinine0.57mg per dl*,* urea 20.5 mg/dl leukocytosis and anaemia haemoglobin 8.3 g/dl platelets’.*	*‘creatinine9.57 mgper dl urea 20.5 mg/dl leukocytosis and anaemia haemoglobin 8.3 g/dl platelets.’* Tortus, Transcript 1	30.4
Diagnosis, pathology and staging	*‘Histology showed invasive ductal adenocarcinoma 50 by 45 mm withnodal metastasis*.*’*	*‘His so showed invasive ductal adenocarcinoma. 50 by 45 mm withno metastases*.*’* Amazon Medical Transcribe, Transcript 28	9.8
Procedure and operative detail	*‘* *undergone an open* *appendicectomy* *and limited right hemicolectomy for a perforated appendix.’*	*‘undergone an openhepatectomyand limited right hemicolectomy for a perforated appendix*.*’ GPT4o Transcribe*,* Transcript 7*	16.1
	*‘* *sleeve* *gastrectomy’*	*‘vasectomy’* Dragon Medical One, Transcript 21	
Medication type and dose	*‘* *fentanyl* *50 micrograms* *’*	*‘fentanyl15mg’* Whisper, Transcript 24	13.2
	*‘* *esomeprazole* *40 mg’*	*‘* *Xarelto* *40 mg’* *Dragon Medical One*,* Transcript 47*	
Complications and clinical course	*‘Respiratory rate 18/min*,* temperature 37 degrees Celsius*,* Oxygen saturation99%on air.’*	*‘Respiratory rate 18/min*,* temperature 37 degrees Celsius*,* Oxygen saturation90%on air.’ Heidi Health*,* Transcript 14*	9.0
Postoperative instructions and follow-up	*‘Dietary caloric restriction with minimally processed foods*,* increased physical activity to150minutes per week with daily walking’*	*‘Dietary caloric restriction with minimally processed foods*,* increased physical activity to15minutes per week with daily walking’ Amazon Medical Transcribe*,* Transcript 73*	1.4
Record context	*‘Within the elevated jejunum on day* *33* *transcatheter arterial chemoembolisation’*	*‘Within the elevated jejunum on day43transcatheter arterial chemoembolisation’ Dragon Medical One*,* Transcript 5*	2.2

Domain category: classification of the error’s clinical context

Reference-transcript: exact excerpt of the spoken content

Transcript-output: ASR output containing the error; the errors are shown in bold

% of Class 3 clinically significant errors: percentage of all Class 3 clinically significant errors attributable to this domain

Table 4 shows median word and character error metrics for each AVT system. Significant differences were seen for WER, DWER, N-DWER, uWER and CER across systems (*P* < 0.001). The experimental (GPT4o-Transcribe-Corrected-5) pipeline consistently achieved lowest median error rates, closely followed by Heidi-Health. In contrast, Amazon Medical Transcribe was consistently worst overall. uWER values exceeded WER for all systems, capturing differences in formatting and punctuation between transcription-outputs and reference-transcripts.

**Table 4:** Comparative error metrics by AVT system

	WER Median (IQR)	DWER Median (IQR)	*N*-DWER Median (IQR)	uWER Median (IQR)	CER Median (IQR)
Amazon Medical Transcribe	14.89 (11.81, 18.35)	24.03 (17.68, 28.58)	16.56 (13.24, 20.96)	32.44 (27.43, 37.41)	5.06 (3.96, 6.66)
Dragon Medical One	12.82 (10.96, 15.84)	10.71 (7.79, 14.49)	14.60 (11.88, 17.89)	30.43 (28.56, 33.94)	4.87 (4.06, 6.29)
GPT4o Transcribe	9.27 (7.08, 11.84)	19.40 (13.14, 24.37)	9.62 (7.79, 13.21)	16.32 (12.87, 18.81)	2.74 (2.16, 3.57)
GPT4o Transcribe Corrected-5	4.05 (3.00, 5.46)	3.60 (1.63, 5.67)	4.15 (3.22, 5.42)	9.90 (7.72, 11.85)	1.43 (1.01, 1.99)
Heidi-Health	5.44 (4.12, 7.75)	5.67 (2.78, 9.26)	5.75 (4.31, 8.49)	14.25 (11.21, 18.39)	1.98 (1.35, 2.98)
Speechmatics enhanced	9.31 (7.38, 11.97)	13.14 (8.98, 16.07)	10.03 (7.89, 13.44)	22.91 (19.74, 28.27)	2.36 (1.88, 3.21)
Tortus	11.51 (9.28, 14.57)	19.36 (13.89, 23.64)	12.67 (10.18, 16.11)	23.26 (20.19, 26.49)	4.40 (3.40, 5.69)
Whisper	11.73 (9.16, 13.79)	21.50 (16.58, 25.83)	12.72 (9.81, 15.44)	19.17 (16.19, 23.08)	3.61 (2.81, 4.56)
P value	< 0.001^a^	< 0.001^a^	< 0.001^a^	< 0.001^a^	< 0.001^a^

*ASR* automatic speech recognition, *CER* character error rate, *DWER* domain word error rate, *IQR* interquartile range, *N-DWER *non-domain word error rate, *uWER *unnormalized word error rate, *WER *word error rate

^a^ Wald-type test for overall differences from quantile regression

Post hoc pairwise comparisons using the GPT4o-Transcribe-Corrected-5 ASR–LLM pipeline as the reference showed considerably higher error rates for all other AVT across all transcription metrics (Supplementary Table S6) with the largest differences observed for Amazon Medical Transcribe and Dragon Medical One.

Table 5 compares within-system performance variability between DWER (medical) and N-DWER (general language) terminologies. For most systems, DWER differed significantly from N-DWER, consistent with AVT having greater difficulty transcribing domain terminology. Interestingly, Dragon Medical One showed the opposite pattern, exhibiting stronger performance and transcription of medical terminology relative to general language. GPT4o-Transcribe-Corrected-5 and Heidi-Health showed no significant difference between DWER and N-DWER (Figure S3).

**Table 5:** Performance variability between DWER versus N-DWER (Wilcoxon Signed-Rank Test)

Amazon Medical Transcribe	*P* value^a^	Effect size
	< 0.001^a^	0.331
Dragon Medical One	< 0.001^a^	-0.332
GPT4o Transcribe	< 0.001^a^	0.405
GPT4o Transcribe Corrected-5	0.31^a^	-0.103
Heidi-Health	0.28^a^	-0.105
Speechmatics enhanced	0.04^a^	0.141
Tortus	< 0.001^a^	0.378
Whisper	< 0.001^a^	0.395

*DWER* domain word error rate, *N-DWER *non-domain word error rate

^a^ Bonferroni adjusted (x8)

Lexical and semantic similarity of AVT transcription-outputs compared to ground truth reference-transcripts is shown in Table 6. ROUGE score measures overall lexical similarity, BERT and BART scores measure semantic accuracy. Significant between-system differences were also observed for ROUGE, BERT, and BART metrics (*P* < 0.001). GPT4o-Transcribe-Corrected-5 achieved the highest similarity to written reference-transcripts, followed by Heidi-Health. ROUGE-1, ROUGE-2 and ROUGE-L individual scores are reported in Supplementary Table S7 and average ROUGE scores are shown in Figure S4. Post hoc comparisons for lexical and semantic outcomes (Supplementary Table S8) showed significantly poorer lexical and semantic similarity for all other systems relative to GPT4o Transcribe Corrected 5 (*P* < 0.001).

**Table 6:** Lexical and semantic similarity metrics and hallucinations by AVT system

	ROUGE Median (IQR)	BERT Median (IQR)	BART Median (IQR)	Hallucinations *n* (%)
Amazon Medical Transcribe	0.847 (0.817, 0.874)	0.854 (0.844, 0.871)	-1.243 (-1.495, -1.070)	0 (0%)
Dragon Medical One	0.871 (0.849, 0.893)	0.813 (0.804, 0.826)	-0.992 (-1.193, -0.874)	0 (0%)
GPT4o Transcribe	0.900 (0.878,0.927)	0.927 (0.904, 0.943)	-0.771 (-0.931, -0.683)	0 (0%)
GPT4o Transcribe Corrected-5	0.963 (0.947, 0.973)	0.962 (0.952, 0.974)	-0.361 (-0.418, -0.324)	0 (0%)
Heidi-Health	0.942 (0.921, 0.961)	0.943 (0.924, 0.958)	-0.467 (-0.536, -0.389)	1 (1%)
Speechmatics enhanced	0.897 (0.868, 0.919)	0.903 (0.888, 0.924)	-0.687 (-0.795, -0.580)	0 (0%)
Tortus	0.870 (0.840, 0.886)	0.901 (0.886, 0.920)	-0.983 (-1.136, -0.862)	0 (0%)
Whisper	0.880 (0.856, 0.897)	0.907 (0.892, 0.923)	-0.974 (-1.114, -0.835)	5 (5%)
P value	< 0.001^a^	< 0.001^a^	< 0.001^a^	0.001^b^

^a^ Wald-type test for overall differences from quantile regression

^b^ from Fisher’s exact test

*IQR* interquartile range, *ROUGE *recall-oriented understudy for gisting evaluation

Hallucinations did occur in outputs from Whisper (*n* = 5) and Heidi-Health (*n* = 1) (Supplementary Table S9) but none were observed for the remaining systems.

Supplementary Table S10 presents Domain Term Missed Ratios for selected SNOMED-CT terms, alongside representative mistranscriptions observed across the AVT tested. These examples illustrate the domain-term errors that contribute to higher DWER than N-DWER observed for most AVT (Table 5).

## Discussion

Accurate clinical documentation is critical for patient safety in surgery, with detail regarding diagnostics, operative-procedures, medication and postoperative instructions directly influencing outcomes and continuity of care. Errors in any of these domains can have clinical consequences, even if the clinical entry reads with seeming fluidity and plausibility. AVT are increasingly being promoted as tools to maximise clinical efficiency and mitigate documentation burden associated with EHRs. In essence, removing the need for a direct physical link between clinician and computer keyboard during the input of clinical information. However, these systems need to be accurate and avoid the introduction of errors, and the current evidence base remains limited [35]. AVT typically rely on ASR to convert dictation or consultation audio into a transcript, which a LLM then structures into a note summary or letter, according to the desired user-template. The efficacy of AVT relies upon fidelity of the transcript-output to the audio-recording and technical reliability of the LLM summarisation. At the simplest level, these systems scribe dictated input from the clinician into a written clinical record, and it is this transcriptional and clinical accuracy that was tested here, rather than LLM summarisation or the full ambient workflow.

As tools for transcription of the surgical record, a key question for AVT is how often a transcription contains at least one mistranscription that is clinically significant. This study provides a comprehensive evaluation of accuracy relating to eight AVT utilising ASR and advocated for use within surgery, focusing on error analysis as well as secondary outcomes involving transcriptional, lexical and semantic accuracy. All systems investigated produced transcripts containing at least one clinically significant Class 3 error, confirming that despite low WER for the best-performing systems, their outputs still require careful verification. Manual error analysis was imperative to understand the type and nature of errors that occurred. We applied a modified Kanal error analysis framework to identify Class 3 errors, which represent subtle changes in meaning in a way that is not obvious [26]. This showed that clinically significant errors persisted across systems, with examples including reversed meaning of comorbidities, distortion of laboratory results, altered interpretation of pathology, documentation of incorrect procedures, medication-type, dose and unit errors, and changes to observations and postoperative instructions. These highlight a specific vulnerability in surgical documentation, because unlike linguistic errors that remain obvious on re-reading, subtle numerical substitutions are harder to detect and can carry disproportionate clinical risk. Numeric values have been shown to be particularly difficult for ASR due to phonetic similarities (‘fifteen’ versus ‘fifty’) and limited semantic context, which forces the model to rely on acoustic cues [36, 37]. However, human written clinical notes can also contain errors and omissions [38, 39] and error rates of 1.47% for hallucinations and 3.45% for omissions using LLM summarisation is below previously reported human note-taking rates [40]. A concern with AVT is that note summarisation adds another point at which errors can be introduced; in an end-to-end pipeline, both transcription and summarisation errors can compound, increasing the overall opportunity for clinically meaningful errors.

Accuracy varied substantially across commercial products, API-accessed ASR systems and the experimental pipeline investigated. Whilst continued technological improvement is inevitable, achieving consistent and reliable transcription of medical information and terminology is currently challenging [20, 41, 42]. Here, the two-stage ASR–LLM experimental pipeline (GPT4o-Transcribe-Corrected-5) and Heidi-Health commercial system both demonstrated high transcription accuracy with WER < 5.5%. To provide context, WER for professional transcription of conversational language has been reported at 5.9% [43]. Although this benchmark is not directly comparable to a surgical dataset, it represents a useful reference value for scale, with the best-performing systems achieving median WER below this level, but performance of other systems substantially worse. For clinical use, this means that for a typical surgical transcript dictated in this study, a WER = 4.1% corresponds to thirteen corrections per transcript, whereas a WER = 14.9% necessitates approximately fifty. The best performing systems reduce but do not eliminate the requirement for transcript editing; the poorest performing risk merely shifting the workload from dictation to significant post-transcription correction.

Whilst not assessing clinical relevance directly, WER remains a useful metric for benchmarking AVT, but a low WER does not define safety, and it is difficult to interpret without understanding where errors occur. For five of the eight systems, medical terminology was transcribed less accurately than general language, consistent with increased difficulty handling technical language. Dragon Medical One was a notable exception, exhibiting the reverse pattern, consistent with its status as a product optimised for medical dictation. However, the bias towards medical terminology came at the cost of performance on general language. In contrast, Heidi-Health and the two-stage ASR-LLM pipeline showed no meaningful performance difference between general and specialist terminology. The Domain Term Missed Ratio helps illustrate the type of errors occurring, with even common clinical terms being replaced by phonetically similar higher probability general language substitutions, or into familiar clinical terminology with reversed meaning (for example, hypo- versus hyperthyroidism). This emphasises that whilst domain training improves the recognition of medical terminology, it does not eliminate the risk of errors that are lexically valid but semantically incorrect.

To assess transcript fidelity beyond error metrics, we used ROUGE (lexical overlap) [32], BART and BERT (semantic similarity) scores [33, 34]. These metrics closely mirrored the rankings associated with transcription errors. However, the exception was again Dragon Medical One; which despite relatively strong performance on medical terminology recorded the lowest BERT score, indicating that recognition of clinical terminology does not necessarily preserve broader semantic integrity (Table 6).

Hallucinations are distinct from mistranscriptions because they introduce content absent from the audio, which can represent a significant quality and safety issue. Here, Hallucinations were uncommon and confined to Whisper and Heidi-Health, ranging from extraneous inserts, ‘Thank you for watching!’ and website-links, to plausible distortions which might alter clinical interpretation. Although no Hallucinations were observed for the remaining systems, this should not be interpreted as evidence that they cannot occur.

The subtlety of errors remains a key concern when using AVT for clinical documentation. When a system has worked well, it becomes easier to defer to its output, particularly in a clinical environment of high cognitive load and time pressure. Indeed, clinical decision-support literature has shown that automation can improve overall performance whilst introducing new ways for errors to occur through over-reliance and reduced vigilance [44]. In AVT, risk can be amplified by the appearance of a polished transcript. For example, we observed a substitution error in which 300 mg aspirin was replaced with 75 mg; both doses being used for different clinical indications making the error plausible and easy to miss. Paradoxically, increased coherence of the note may lower a clinician’s index of suspicion during review and increase safety risks. This risk is not uniform across dictation length. For four systems, odds of a clinically significant error increased significantly per additional 100 words, implying that for these systems, longer dictations carry both greater risk and burden for clinician review.

The experimental pipeline demonstrated that LLM-processing of ASR input was most effective when the error could be resolved from surrounding context. For some dosing-unit errors, the medication and dose in the raw ASR output provided sufficient context for the LLM to correct the unit. In contrast, errors related to quantitative values persisted as the correct values, relationships and omitted details could not be reliably inferred from the raw ASR output. Likewise, despite a significant reduction in clinically significant errors and aggregate accuracy shown in other studies [21, 16], this study highlights that the potential for new errors, with four errors being newly introduced by the LLM in the experimental pipeline, highlighting the need for vigilance as errors reduce in frequency but still persist.

Considering the effects of latency in the LLM-processing step, in the earlier orthodontic study, a pilot of 20 recordings showed that the LLM correction had a median processing time of 3.92 s (IQR 2.85–4.53) [16], which is unlikely to be consequential for overall documentation time. However, for deployment considerations this does introduce another API request and additional cost. A recent study in general practice showed that ambient scribing reduced documentation time by 42.7 s but consultation time did not change significantly [45]. While the observed reduction in clinically significant errors supports the LLM-processing step, its real-world value should be judged by the complete ambient documentation workflow.

Medicolegally, clinicians remain responsible for accuracy of the clinical record [9] and NHS England [10] emphasises clinician review and ongoing monitoring through audit. This aligns with established safety practice within surgery, where structured checklists such as the WHO Surgical Safety Checklist [46] and National Safety Standards for Invasive Procedures [47] are used to reduce low-frequency, high-consequence preventable failures in surgical units [48]. For clinicians, the adoption of AVT must be paired with explicit checklist behaviour focused on high-risk content including medications, doses/units, laboratory values, procedures and laterality. Further work will need to involve training clinicians to work with AVT and optimise the careful balance between automation and clinician oversight so clinicians do not become ‘liability sinks’ for AI [49, 50].

Future work should move beyond simply asking clinicians to detect these errors during review and instead ask how interaction with the EHR can be designed to make highlighting these errors easier and more reliable? Deeper integration of AVT with the EHR may allow draft notes to draw on existing patient context such as prior records, medications, allergies, laboratory results with this context, supporting verification by flagging discordance between dictated content and existing structured data rather than an LLM inferring unsupported values. However, this also introduces opportunities for errors such as the retrieval of outdated information, propagation of previous documentation errors and omission of relevant context. Research must therefore include developers, clinicians, EHR vendors to take a systems engineering approach in the co-design of AVT-EHR integration in order to improve accuracy, patient safety and reduce documentation burden. This also must be done with careful consideration given to ensuring patient data is kept both private and secure [51].

This study has several limitations. Firstly, the surgical case-reports comprised narratives read by a single speaker limiting representativeness and potentially introducing speaker-specific bias, with the results likely underestimating error-rates compared with busy clinical practice which involves accent variation, multiple-speakers, interruptions and background noise. Deployment of these tools should ensure that they are accurate for all patients and staff (for example, higher error rates have been reported for Black patients compared with White) [15]. Secondly, we focused on the speech-to-text component rather than end-to-end ambient scribing of live consultations. Therefore, we did not capture additional failure modes introduced by LLM note summarisation. Familiarity with publicly available case-report wording may have also favoured textual reconstruction for the LLM-processing step. The generalisability of these findings across all specialities of medicine is limited where vocabulary may differ and these findings should be interpreted as specific to narrated clinical surgery under controlled conditions. Finally, while the primary outcome focused on Class 3 errors where the meaning was not obvious, Class 0–2 errors should also be interpreted in the future as they may increase editing burden and reduce readability, making the intended meaning more difficult for clinicians to interpret under time pressure.

Future evaluations should focus on prospective assessment in clinical practice (outpatients, ward rounds, theatre) and end-to-end evaluations of AVT, assessing not only transcripts but the final note, and measuring editing burden (time to review notes and clinically significant errors) rather than relying on accuracy-metrics alone. In parallel, work should focus on practical mitigations to prevent clinical documentation errors that may lead to harm, such as user interfaces that draw attention to high-risk content such as medications, doses/units, laboratory values, laterality and procedures.

## Conclusions

In this controlled single-speaker investigation, the accuracy of AVT speech-to-text function varied substantially between systems. Clinically significant errors were identified for all systems tested, affecting the quality and safety of transcripts, with potential for patient harm. The safety and effectiveness of these systems in clinical practice requires further evaluation, including the role of the clinician in verifying transcription outputs.

## Supplementary Information

Below is the link to the electronic supplementary material.

Supplementary Material 1

## Data Availability

Data and analysis materials are made available at Zenodo https://doi.org/10.5281/zenodo.18209141
