# Supplementary material for: A Controlled Single-Speaker Evaluation of Ambient Voice Technologies’ Speech-to-Text Function Using Narrated Surgical Case-Reports
Source: J Med Syst. 2026 Oct 9;50(1):145. doi: 10.1007/s10916-026-02471-5 (PMC13652911; doi:10.1007/s10916-026-02471-5)
Supplement: Supplementary file 1 [file 10916_2026_2471_MOESM1_ESM.docx]

**Supplementary material**

**Supplementary methods**

Dataset creation

A corpus of 100 written case-reports derived from open-access surgical journals indexed in PubMed Central was used for the investigation. To identify reports, PubMed Central was searched using the MeSH heading “Surgical Procedures, Operative” combined with a broad set of operative keywords in the title and abstract (e.g., *surg*, *operat*, resection, repair, laparoscop*, anastomos*, graft*, fixation, bypass, stoma, transplant*). Words suggesting novelty (e.g. “rare” “first report”, “novel”) were excluded from the search strategy to focus on case reports detailing generalisable surgical procedures. For each case-report, we selected a case-summary describing patient presentation, operative management and postoperative course. All articles were published under Creative Commons Licenses (CC-BY, CC BY-NC or CC BY-NC-SA) permitting textual reuse for research. These case-summaries were collated into a reference library and served as the written ground truth reference-transcript for all subsequent analyses of transcription accuracy.

Each reference-transcript was then narrated by a single individual (RO) in a closed-environment at King’s College London using a RODE NT-USB (RØDE, Australia) mini-microphone connected via USB-C to a MacBook Air M3 and recorded in Audacity for Mac (version 3.7.1). Audio files were recorded in [.wav] format at 16-bit depth and 44.1kHz to ensure high audio quality. The recorded audio totalled 4 hours 38 minutes. All systems were tested between 22^nd^ October and 1^st^ November 2025. Each of the (n=100) individual [.wav] audio-recordings was passed through the 3 commercial transcription-systems: (Dragon Medical One, Heidi-Health, Tortus) and 4 standalone ASR systems accessed through speech-to-text Application Programming Interfaces (Speechmatics Enhanced, Amazon Medical Transcribe, Whisper, GPT4oTranscribe) being investigated, producing seven outputs per recording. To run audio files through the systems, for Heidi-Health and Tortus the [.wav] file was directly uploaded and the resulting word-for-word transcription stored; for Dragon Medical One (version 2025.3, build 25.3.60179.0), a VB-CABLE Virtual Audio Device was used to play back the [.wav] files at 44.1kHz so quality was preserved and the software recognised them as the input. Audio-recordings in [.wav] were processed through speech-to-text APIs of Amazon Medical Transcribe (‘Primary Care, Dictation’), Whisper (‘whisper-1’) and GPT4o Transcribe OpenAI (‘gpt-4o-transcribe’), Speechmatics Enhanced (API v2). All AVT were used with their default parameters. For the experimental two-stage ASR-Large Language Model (LLM) pipeline incorporating GPT4o Transcribe, transcription-outputs underwent an LLM generative error-correction step, with each output sent to the Chat completions endpoint (v1/chat/completions) of the OpenAI API using the model gpt-5-2025-08-07 as text. Temperature was not configurable and was therefore not supplied. The full prompt used can be seen in Supplementary Figure S1. This produced an eighth transcription-output for each recording, leading to a total of (n=800) system transcription-outputs for investigation.

The transcription-outputs generated were collated into Microsoft Excel for Mac (version 16.92) (Microsoft Corp, Burlington, MA, USA) as individual files for subsequent evaluation.

The ground truth written reference-transcripts were then compared against the system transcription-outputs using Visual Studio Code for Mac (version 1.95, Microsoft Corp, Burlington, MA, USA) with Python (<http://www.python.org/>, version 3.11). The evaluation metrics were calculated using the packages:

pandas (version 2.3.3 <https://pandas.pydata.org>),

torch (version 2.6.0 <https://pytorch.org/>),

transformers (version 4.57.1, <https://github.com/huggingface/transformers>),

rouge_score (version 0.1.2 <https://github.com/google-research/google-research/tree/master/rouge>),

bert_score (version 0.3.13 <https://github.com/Tiiiger/bert_score>),

jiwer (version 3.1.0 <https://github.com/jitsi/jiwer>) and

openpyxl (version 3.1.5 <https://openpyxl.readthedocs.io>).

Error Analysis

Transcription errors identified by the jiwer package during uWER calculation, were categorised using a modified version of Kanal’s error analysis [1]. Class 0 errors are formatting errors which are grammatically correct and do not change the meaning of the text; Class 1 errors are grammatically incorrect but do not change the meaning of the text; Class 2 errors produce a change in meaning, but the error is obvious and can be easily identified; Class 3 errors change the meaning in a way that is not obvious upon immediate inspection. Class 3 errors were then categorised according to whether they were clinically significant and could lead the reader to misunderstand the clinical meaning, potentially impacting patient care. [2] Two reviewers manually annotated errors and assessed clinical-significance of each Class 3 error. Any queries or discrepancies were resolved by a third reviewer. For Class 3 errors judged to be clinically significant, errors were then categorised by error domain and potential harm-severity. To assess the severity of these errors, considering the plausible consequence if the error was propagated uncorrected and acted upon, severity was rated using a four level scale aligned with NHS England levels of harm. The degree of potential harm was recorded as Level 1 (low), Level 2 (moderate), Level 3 (severe/serious deterioration), or Level 4 (death) [3].

Word Error Rate

WER quantifies transcription accuracy by comparing the number of word substitutions, insertions and deletions to the total number of words in the written reference-text after normalisation.

$$\mathrm{WER}=\frac{S+I+D}{N}\times100\%$$

Where S=number of substitutions; I=insertions; D=deletions; and N=total words in the reference text. Although WER provides an overall measure of performance, it may insufficiently capture domain-specific nuances. The normalised WER is reported.

Domain Word Error Rate

DWER extends WER by focusing on errors associated with specialised terminology, offering a more precise indicator of how effectively an ASR system handles the technical vocabulary required. In this study, we calculated DWER by focusing on tokens from a curated domain-specific vocabulary derived from Systematized Nomenclature of Medicine Clinical Terms SNOMED CT International Edition, English Description snapshot (release 2025-08-01), a structured clinical vocabulary used in electronic health records. DWER provides a more targeted indicator of how effectively an ASR system handles the specialised vocabulary.

$$\text{DWER}=\frac{S_{d}+I_{d}+D_{d}}{N_{d}}\times100\%$$

Where *S_d_=*number of substitutions made on domain tokens; *I_d_=*number of insertions made on domain tokens; *D_d_=*number of deletions made on domain tokens; and *N_d_=*total number of domain tokens in the reference text.

Non-Domain Word Error Rate

Non-Domain WER (N-D WER) is computed by excluding all domain-specific terms. This metric evaluates the system performance on general language tokens, providing insight into accuracy outside the specialised terminology.

$$N-D \text{WER}=\frac{S_{nd}+I_{nd}+D_{nd}}{Nnd}\times100\%$$

Where *S_nd_=*number of substitutions made on non-domain tokens; *I_nd_=*number of insertions made on non-domain tokens*; D_nd_=*number of deletions made on non-domain tokens*;* and *N_nd_=*total number of non-domain tokens in the reference text.

Unnormalised Word Error Rate

The unnormalised WER (uWER) is calculated without normalisation, hence penalising formatting differences, such as punctuation variations and alternative transcriptions of numbers (e.g. ‘2’ versus ‘two’ or ‘II’). Consequently, uWER scores tend to be higher than WER scores, a smaller gap between WER and uWER reflects closer adherence to the exact reference formatting.

Character Error Rate

A Character Error Rate (CER) is similar to WER but operates at a finer level of granularity by quantifying transcription errors on a per-character basis rather than per word.

$$CER=\frac{S+I+D}{N}\times100\%$$

Effect Size

To compare the performance of each tool on domain-specific versus non-domain text, we conducted Wilcoxon signed-rank tests. We then calculated effect sizes (*r*) from the test statistic using the formula:

$$r=\frac{Z}{\surd(4N)}$$

Where *Z* is the Wilcoxon *z*‐score, and *N* is the total number of observations. A larger *r*‐value indicates greater divergence between domain‐specific and non‐domain performance (i.e. the system struggles more with the clinical terminology relative to general language); whereas a smaller *r*‐value, suggests more consistent performance across both types of content.

Lexical accuracy

The word-level precision of the transcription-output in comparison to the reference-transcript was calculated using the ROUGE (Recall-Orientated Understudy for Gisting Evaluation) software package for automatic quantitative evaluation of similarity between two texts [4]. Specifically, within this package ROUGE-1 measures the overlap of unigrams (individual words) between generated text and the reference transcription, ROUGE-2 measures the overlap of bigrams (pairs of consecutive words) and ROUGE-L focuses on the longest common subsequence (LCS) to determine how well the order of words in the transcription-output matches the written reference-transcript. An overall ROUGE Score was computed by aggregating ROUGE-1, ROUGE-2 and ROUGE-L.

Semantic similarity

The preservation of meaning and contextual integrity of the transcription-output in comparison to reference-transcript was calculated using BERT Score and BART Score, two automatic evaluation metrics for text generation. BERT Score computes a similarity score for each token in the candidate sentence with each token in the reference sentence [5]. This gives an insight into the token-level alignment between texts; higher scores indicate stronger semantic similarity between the transcription-output and reference-transcript. In contrast, BART Score employs a pre-trained BART model to estimate the likelihood of a reference given a candidate. [6]. This metric captures the overall fluency of the candidate text relative to the reference: higher (or less negative) BART scores suggest that the candidate text is semantically closer to the reference. These evaluation metrics, commonly used in summarisation tasks, provide complementary perspectives on semantic accuracy.

Hallucinations

A manual review of each transcription-output was undertaken to identify ‘hallucinations’, defined as the introduction of incorrect or misleading information by the ASR system not present in the audio. This was done manually, with the full audio-recording listened to whilst reading the transcription-output, flagging any extraneous words or phrases that were not actually spoken. These flagged segments of text were recorded in Microsoft Word for Mac. A hallucination rate was then calculated for each system as the proportion of transcription-outputs containing at least one hallucination relative to the total number of reference-transcripts.

Domain-Term Missed Ratio Analysis

To evaluate performance specifically on what terms are missed, a Domain Term Missed Ratio was calculated. This analysis focused on domain tokens drawn from a dictionary derived from the Systematized Nomenclature of Medicine Clinical Terms structured clinical health terminology product for electronic health records and supplemented with additional terms verified by clinicians in the reference-transcripts [7]. Each transcription-output was examined to determine whether these tokens (including recognised synonyms) were correctly transcribed. The missed ratio represents the proportion of these domain tokens that were not successfully reproduced by the ASR system, with higher values indicating poorer performance.

$$Missed Ratio=\frac{Number of domain tokens missed in ASR output}{Total number of domain tokens in reference}\times100\%$$

Example mistranscriptions were then manually reviewed to highlight specific instances of domain-token errors.

Normalisation

There are multiple equally valid ways to express the same concept in written text; for instance, ‘millimetres’ can appear as ‘mm’ and ‘three out of four’ as ‘3/4’. These superficial variations can inflate error metrics when assessing ASR outputs. To minimise this and ensure fair comparisons across systems, we applied a text-normalisation step to both the written reference-transcript and transcription-output, focusing on formatting and stylistic differences rather than altering the underlying content, in line with common practice for WER evaluation [8]. Normalisation practices were adapted for the nature of the terms and nomenclature used in surgery. By prioritising consistent transcription of terms, errors were penalised only for genuine mistranscription.

Specifically, normalisation involved converting transcriptions into a standardised format, enabling comparable WER to be calculated across systems. Briefly, steps included case standardisation, converting all text to lowercase and expanding common contractions (informal contractions such as ‘there’s’ were expanded to their full forms, e.g. ‘there is’); numeric formatting, including removing of commas within numbers to standardise numerical expressions and consistent handling of simple number words, fractions and decimals; punctuation consistency, all unnecessary punctuation was removed including periods not followed by digits and apostrophe consistency implemented, unit standardisation, with measurements standardised to abbreviations, such as ‘millimetres’ converted to ‘mm’ and “grams” to “g”.

**Supplementary Figure S1	System prompt used for LLM generative error correction.**

**Supplementary Figure S2	Severe clinically significant errors by domain category**. Bars show the number of clinically significant Class 3 errors rated as severe (potential harm Level 3) or death (potential harm Level 4) within each domain category

**Supplementary Figure S3	Median DWER versus N-DWER by AVT-system.** Bars show the median error rate and whiskers indicate the interquartile range (IQR: 25^th^-75^th^ percentile).

**Supplementary Figure S4	Average ROUGE metrics for AVT system tested**

**Supplementary Table S1**	Inter-rater reliability in error analysis

| **Variable** | **Agreement** | **Expected agreement** | **Kappa**  **(95% Confidence interval)** |
| --- | --- | --- | --- |
| Kanal Class error categorisation | 95.69% | 77.79% | 0.806 (0.799, 0.812) |
| Proportion of clinically significant errors | 96.61% | 56.03% | 0.923 (0.903, 0.939) |

**Supplementary Table S2**	Effect of word length count (per 100 additional words) for each AVT-system

|  | **Total errors (n)** | **Number of Class 3 clinically significant errors (n)** | **Transcript-outputs with at least 1 clinically significant error, % [95% CI]** | **Odds Ratio (95% CI)** | **P*** |
| --- | --- | --- | --- | --- | --- |
| Amazon Medical Transcribe | 4,147 | 145 | 68.0 [58.1-76.5] | 2.85 (1.45-5.60) | 0.01 |
| Dragon Medical One | 3,818 | 177 | 68.0 [58.1-76.5] | 2.98 (1.55-5.71) | 0.007 |
| GPT4o Transcribe | 2,811 | 89 | 53.0 [43.1-62.7] | 2.65 (1.32-5.32) | 0.04 |
| GPT4oTranscribeCorrected-5 | 1,248 | 42 | 30.0 [21.7-39.8] | 1.59 (0.81-3.16) | 1.00 |
| Heidi-Health | 1,528 | 89 | 54.0 [44.0-63.7] | 1.81 (0.96-3.39) | 0.45 |
| Speechmatics enhanced | 2,850 | 87 | 48.0 [38.2-57.9] | 1.99 (1.05-3.76) | 0.24 |
| Tortus | 3,417 | 143 | 62.0 [52.0-71.1] | 3.34 (1.61-6.90) | 0.008 |
| Whisper | 3,337 | 77 | 44.0 [34.5-54.0] | 1.51 (0.83-2.75) | 1.00 |

CI, confidence interval

^*^ Bonferroni correction applied

**Supplementary Table S3**	Comparison of transcript-outputs with at least 1 clinically significant error between GPT4oTranscribe and GPT4oTranscribeCorrected

| **System** | **Error rate** | **Odds ratio** | **95% Confidence interval** | **P** |
| --- | --- | --- | --- | --- |
| GPT4oTranscribe | 53% | Reference |  |  |
| GPT4oTranscribeCorrected-5 | 30% | 0.38 | 0.25-0.58 | <0.001 |

**Supplementary Table S4**	Qualitative analysis of clinically significant errors by type and outcome following LLM-processing of raw GPT4o Transcribe ASR output

| **Error family** | **Error category** | **Resolved, n** | **Remained clinically significant, n** | **Newly clinically significant, n** | **Total, n** | **Example** |
| --- | --- | --- | --- | --- | --- | --- |
| Clinical terminology and context | Raw clinical term which is inconsistent with context | 21 | 0 | 0 | 21 | *Resolved example* **Raw:** ‘methicillin-sensitive Staphylococcus aureus … flucocytosine 2 g four times daily’  **LLM-processed:** ‘flucloxacillin 2 g four times daily’ **Reference:** ‘flucloxacillin 2 g four times daily’ |
|  | Raw clinical term which is plausible but incorrect | 11 | 7 | 0 | 18 | *Remained clinically significant example* **Raw:** ‘open hepatectomy and limited right hemicolectomy for a perforated appendix’ **LLM-processed:** ‘open hepatectomy and limited right hemicolectomy’  **Reference:** ‘open appendicectomy and limited right hemicolectomy’ |
|  | Internally inconsistent clinical detail | 2 | 5 | 0 | 7 | *Remained clinically significant example* **Raw:** ‘pT4b … and 1 M0, pStage IIIC’ **LLM-processed:** ‘pT4b … N2 M0, pStage IIIC’ **Reference:** ‘pT4b … N1 M0, pStage IIIC’ |
| Quantitative detail and units | Incorrect quantitative detail | 9 | 12 | 0 | 21 | *Remained clinically significant example* **Raw:** ‘6%’ **LLM-processed:** ‘6%’  **Reference:** ‘60%’ |
|  | Incorrect units | 4 | 1 | 0 | 5 | *Resolved example* **Raw:** ‘dobutamine 7.5 mg/kg/min’ **LLM-processed:** ‘dobutamine 7.5 micrograms/kg/min’ **Reference:** ‘dobutamine 7.5 micrograms/kg/min’ |
| Meaning and completeness | Reversed meaning | 3 | 8 | 0 | 11 | *Resolved example* **Raw:** ‘intra-abdominal abscess managed inappropriately’ **LLM-processed:** ‘intra-abdominal abscess managed appropriately’ **Reference:** ‘intra-abdominal abscess managed appropriately’ |
|  | Omitted or insufficiently specific detail | 1 | 4 | 0 | 5 | *Remained clinically significant example* **Raw:** ‘left nephrectomy’ **LLM-processed:** ‘left nephrectomy’ **Reference:** ‘left nephroureterectomy’ |
| Source faithfulness | Unsupported addition or reinterpretation | 0 | 1 | 4 | 5 | *Newly clinically significant example* **Raw:** ‘left renal malformation tumour’ **LLM-processed:** ‘left renal malignant tumour’ **Reference:** ‘left renal malformation tumour’ |
| **Total** |  | **51** | **38** | **4** | **93** |  |

**Supplementary Table S5	Potential harm severity count by AVT system**

|  | **Total (n)** | **Potential harm Level-1 (Low)** | **Potential harm Level-2 (Moderate)** | **Potential harm Level-3 (Severe)** | **Potential harm Level-4 (Death)** |
| --- | --- | --- | --- | --- | --- |
| Amazon Medical Transcribe | 145 | 69 | 69 | 7 | 0 |
| Dragon Medical One | 177 | 77 | 84 | 16 | 0 |
| Tortus | 143 | 54 | 82 | 6 | 1 |
| GPT4o Transcribe | 89 | 40 | 45 | 3 | 1 |
| Speechmatics enhanced | 87 | 38 | 46 | 3 | 0 |
| Heidi-Health | 89 | 31 | 51 | 7 | 0 |
| Whisper | 77 | 36 | 34 | 5 | 2 |
| GPT4o Transcribe Corrected-5 | 42 | 13 | 27 | 2 | 0 |

Counts show the number of clinically significant Class 3 errors assigned to each potential harm level (Levels 1-4) for each AVT-system.

**Supplementary Table S6**	Pairwise comparison of AVT system for word and character error outcomes

|  | **WER** | | **DWER** | | **N-D WER** | | **uWER** | | **CER** | |
| --- | --- | --- | --- | --- | --- | --- | --- | --- | --- | --- |
|  | **b (95% CI)** | **P-value*** | **b (95% CI)** | **P-value*** | **b (95% CI)** | **P*-value** | **b (95% CI)** | **P*-value** | **b (95% CI)** | **P*-value** |
| **GPT4o Transcribe Corrected-5** | Reference |  | Reference |  | Reference |  | Reference |  | Reference |  |
| **Amazon Medical Transcribe** | 10.90  (9.81, 12.00) | <0.001* | 20.17  (18.32, 22.03) | <0.001* | 12.40  (11.00, 13.81) | <0.001* | 22.90  (21.70, 24.10) | <0.001* | 3.64  (3.22, 4.06) | <0.001* |
| **Dragon Medical One** | 8.78  (7.85, 9.72) | <0.001* | 7.08  (5.62, 8.54) | <0.001* | 10.44  (9.44, 11.43) | <0.001* | 20.59  (19.54, 21.63) | <0.001* | 3.44  (3.06, 3.82) | <0.001* |
| **GPT4o Transcribe** | 5.20  (4.21, 6.19) | <0.001* | 15.60  (13.57, 17.62) | <0.001* | 5.49  (4.75, 6.22) | <0.001* | 6.44  (5.29, 7.59) | <0.001* | 1.30  (1.05, 1.56) | <0.001* |
| **Heidi-Health** | 1.38  (0.56, 2.19) | 0.007* | 2.05  (0.68, 3.41) | 0.02* | 1.61  (0.80, 2.43) | 0.001* | 4.43  (3.26, 5.60) | <0.001* | 0.56  (0.26, 0.86) | 0.002* |
| **Speechmatics enhanced** | 5.22  (4.31, 6.13) | <0.001* | 9.48  (8.05, 10.91) | <0.001* | 5.87  (5.02, 6.72) | <0.001* | 13.16  (11.75, 14.57) | <0.001* | 0.94  (0.70, 1.18) | <0.001* |
| **Tortus** | 7.48  (6.49, 8.53) | <0.001* | 15.72  (14.05, 17.38) | <0.001* | 8.51  (7.39, 9.63) | <0.001* | 13.45  (12.44, 14.47) | <0.001* | 3.00  (2.62, 3.38) | <0.001* |
| **Whisper** | 7.64  (6.83, 8.44) | <0.001* | 17.79  (16.14, 19.44) | <0.001* | 8.60  (7.68, 9.52) | <0.001* | 9.37  (8.36, 10.38) | <0.001* | 2.18  (1.90, 2.46) | <0.001* |

b, regression coefficient; CER, character error rate; CI, confidence interval; DWER, domain word error rate; N-DWER, non-domain word error rate; uWER, unnormalised word error rate; WER, word error rate

* Bonferroni correction applied

**Supplementary Table S7**	Comparison of different ROUGE metrics by AVT system

|  | **ROUGE-1**  Median  (IQR) | **ROUGE-2**  Median  (IQR) | **ROUGE-L**  Median  (IQR) |
| --- | --- | --- | --- |
| **Amazon Medical Transcribe** | 0.880  (0.857, 0.904) | 0.778  (0.741, 0.819) | 0.876  (0.851, 0.901) |
| **Dragon Medical One** | 0.908  (0.885, 0.926) | 0.808  (0.776, 0.841) | 0.899  (0.880, 0.919) |
| **GPT4o Transcribe** | 0.923  (0.905, 0.943) | 0.855  (0.825, 0.894) | 0.923  (0.904, 0.943) |
| **GPT4o Transcribe Corrected-5** | 0.972  (0.961, 0.980) | 0.943  (0.922, 0.958) | 0.971  (0.959, 0.980) |
| **Heidi-Health** | 0.958  (0.940, 0.971) | 0.915  (0.886, 0.940) | 0.955  (0.937, 0.971) |
| **Speechmatics enhanced** | 0.920  (0.897, 0.939) | 0.854  (0.814, 0.879) | 0.919  (0.894, 0.936) |
| **Tortus** | 0.901  (0.878, 0.914) | 0.809  (0.768, 0.831) | 0.898  (0.873, 0.913) |
| **Whisper** | 0.907  (0.890, 0.922) | 0.827  (0.793, 0.851) | 0.904  (0.885, 0.921) |
| P-value | <0.001* | <0.001* | <0.001* |

* Wald-type test for overall differences from quantile regression

IQR, interquartile range; ROUGE, recall-oriented understudy for gisting evaluation

**Supplementary Table S8**	Post hoc pairwise comparison of AVT system for lexical and semantic outcomes

|  | **ROUGE** | | **ROUGE-1** | | **ROUGE-2** | | **ROUGE-L** | | **BART** | | **BERT** | | |
| --- | --- | --- | --- | --- | --- | --- | --- | --- | --- | --- | --- | --- | --- |
|  | **b (95% CI)** | **P-value*** | **b (95% CI)** | **P-value*** | **b (95% CI)** | **P-value*** | **b (95% CI)** | **P-value*** | **b (95% CI)** | **P-value*** | **b (95% CI)** | **P-value*** |  |
| **GPT4o Transcribe Corrected-5** | Reference |  | Reference |  | Reference |  | Reference |  | Reference |  | Reference |  |  |
| **Amazon Medical Transcribe** | -0.12  (-0.13, -0.10) | <0.001* | -0.09  (-0.10, -0.08) | <0.001* | -0.17  (-0.18, -0.15) | <0.001* | -0.10  (-0.11, -0.08) | <0.001* | -0.88  (-0.96, -0.80) | <0.001* | -0.11  (-0.11, -0.10) | <0.001* |  |
| **Dragon Medical One** | -0.09  (-0.10, -0.09) | <0.001* | -0.06  (-0.07, -0.06) | <0.001* | -0.14  (-0.15, -0.12) | <0.001* | -0.07  (-0.08, -0.06) | <0.001* | -0.63  (-0.70, -0.56) | <0.001* | -0.15  (-0.15, -0.14) | <0.001* |  |
| **GPT4o Transcribe** | -0.06  (-0.07, -0.06) | <0.001* | -0.05  (-0.05, -0.04) | <0.001* | -0.09  (-0.10, -0.08) | <0.001* | -0.05  (-0.06, -0.04) | <0.001* | -0.41  (-0.46, -0.36) | <0.001* | -0.04  (-0.04, -0.03) | <0.001* |  |
| **Heidi-Health** | -0.02  (-0.03, -0.01) | <0.001* | -0.02  (-0.02, -0.01) | <0.001* | -0.03  (-0.04, -0.02) | <0.001* | -0.02  (-0.02, -0.01) | <0.001* | -0.11  (-0.14, -0.07) | <0.001* | -0.02  (-0.03, -0.01) | <0.001* |  |
| **Speechmatics enhanced** | -0.07  (-0.07, -0.06) | <0.001* | -0.05  (-0.06, -0.04) | <0.001* | -0.09  (-0.10, -0.07) | <0.001* | -0.05  (-0.06, -0.04) | <0.001* | -0.33  (-0.37, -0.28) | <0.001* | -0.06  (-0.06, -0.05) | <0.001* |  |
| **Tortus** | -0.09  (-0.10, -0.09) | <0.001* | -0.07  (-0.08, -0.07) | <0.001* | -0.13  (-0.14, -0.12) | <0.001* | -0.07  (-0.08, -0.07) | <0.001* | -0.62  (-0.67, -0.58) | <0.001* | -0.06  (-0.07, -0.05) | <0.001* |  |
| **Whisper** | -0.08  (-0.09, -0.08) | <0.001* | -0.07  (-0.07, -0.06) | <0.001* | -0.12  (-0.13, -0.10) | <0.001* | -0.07  (-0.07, -0.06) | <0.001* | -0.61  (-0.67, -0.55) | <0.001* | -0.06  (-0.06, -0.05) | <0.001* |  |

b, regression coefficient; CER, character error rate; CI, confidence interval; DWER, domain word error rate; N-D WER, non-domain word error rate; uWER, unnormalised word error rate; WER, word error rate

* Bonferroni correction applied.

**Supplementary Table S9	Hallucinations in AVT system transcription-outputs**

| **ASR system** | **Audio-recording number** | **Reference-transcript** | **Transcription-output** |
| --- | --- | --- | --- |
| **Whisper** | 11 | … His postoperative course was complicated by an abscess. … | … His post-operative course was complicated by a **therapeutic** abscess. … |
| **Whisper** | 18 | … with concurrent sensory and electrophysiological improvement. … | … with concurrent sensory and electrophysiological improvement. **Thank you for watching**! |
| **Whisper** | 40 | … resolution of the fistula with a clean cavity and healthy granulation tissue. | … resolution of the fistula with a clean cavity and healthy granulation tissue. **For more UN videos visit www.un.org** |
| **Whisper** | 44 | …White cell count 23.3 × 10^9/L, 93% neutrophils; total bilirubin 8.4 mg/dL. Blood cultures yielded Peptostreptococcus micros and he improved with ampicillin/sulbactam... | ..white cell count 23.3. **The patient's blood test showed that he had a high BMI of 16.8 kgm2 and febrile with cholangitis,** white cell count 23.3, 95% neutrophils to bilirubin 8.4. Blood cultures yielded peptostreptococcus micros and he improved with ampicillin, so back down… |
| **Whisper** | 75 | … At her most recent annual follow-up she remained well, with … without further abdominal or shunt-related complications. | … At her most recent annual follow up she remained well with … without further abdominal or shunt related complications**. For more information visit www.fda.gov.au** |
| **Heidi-Health** | 11 | … His postoperative course was complicated by an abscess. … | … His postoperative course was complicated by a **sympathetic** abscess. … |

This table presents instances where AVT-system produced hallucinations. The table includes the AVT-system responsible for the hallucination, audio-recording number, written reference-transcript and generated transcription-output.

Hallucinated text within the transcription-output is highlighted in bold.

**Supplementary Table S10	Domain-terms, overall domain-term missed ratio and corresponding mistranscriptions across AVT systems**

| **Domain-term** | **Overall domain-term**  **missed ratio (%)** | **Example mistranscriptions** |
| --- | --- | --- |
| Olmesartan | 87.5 | *'omeprazole', 'omesolterin', 'copitagryl', 'omisaltan', 'omnisultran', 'soltan', 'sultan'* |
| Macroscopy | 87.5 | *'microscopic', 'microscopically', 'microscopy'* |
| Hyperparathyroidism | 75 | *'hyperpyrephoretism', 'hyperpyroidism', 'hyperpyrophilism', 'hyperpyrthyroidism', 'hyperthyroidism', 'theroid'* |
| Omentectomy | 62.5 | *'amenectomy', 'aminectomy', 'amy', 'ectomy', 'emendectomy', 'omentoectomy'* |
| Cephalic | 62.5 | *'catholic'* |
| Oestrogen | 50 | *'eastern', 'in eastern'* |
| Paroxysmal | 50 | *'prigsmal', 'prismal', 'proximal', 'primo'* |
| Ischaemia | 62.5 | *'Asemia', 'Asia', 'bile asemia'* |
| Defecography | 87.5 | *'demography', 'free', 'gray', 'main diffractography', 'main diphography'* |
| Fibromatosis | 75 | *'fatsis', 'firmatosis', 'phrymatosis', 'from ptosis'* |
| Phlegmonous | 68.8 | *‘flaminous’, ‘flimonous’, ‘fomon’, ‘fulminant’, ‘fulminous’* |
| Lithiasis | 55 | *'diphthisis', 'diphthysis', 'diphysis', 'lighthiasis'* |
| Papillotomy | 37.5 | *'ablotomy', 'papillomectomy', 'ploy'* |
| Intrathoracic | 37.5 | *'intraforacic', 'intraphrastic'* |
| Hyperthyroidism | 25 | *'hypothyroidism'* |
| Enteroscopy | 25 | *'endoscopy'* |
| Intraluminal | 20.3 | *'interlaminal', 'interliminal', 'interluminal', 'interluminal tumor', 'luminal', 'mineral'* |
| Histology | 8.6 | *'astrology', 'estology'* |
| Extubated | 4.7 | *'executed', 'is excluded'* |
| Creatinine | 12.5 | *'carotene', 'carotenin', 'carotidine', 'curatinine'* |

Overall domain-term missed ratio (%) is the proportion of times a term was mis-transcribed across all AVT-systems divided by the total number of occurrences of that clinical term across all eight systems. A 100% overall missed ratio indicates all ASR systems failed to transcribe the term correctly

**Supplementary references**

1. Kanal, K. M., Hangiandreou, N. J., Sykes, A. M., Eklund, H. E., Araoz, P. A., Leon, J. A., et al. (2001). Initial evaluation of a continuous speech recognition program for radiology. *J Digit Imag, 14* (1), 30-37, doi:10.1007/s10278-001-0022-z.

2. Chang, C. A., Strahan, R., & Jolley, D. (2011). Non-clinical errors using voice recognition dictation software for radiology reports: a retrospective audit. *J Digit Imag, 24* (4), 724-728, doi:10.1007/s10278-010-9344-z.

3. NHS England (2024). Policy guidance on recording patient safety events and levels of harm. <https://www.england.nhs.uk/long-read/policy-guidance-on-recording-patient-safety-events-and-levels-of-harm/>. Accessed 14th December 2025.

4. Lin, C.-Y. ROUGE: A Package for Automatic Evaluation of Summaries. In *Text Summarization Branches Out, Barcelona, Spain, 2004* (pp. 74-81): Association for Computational Linguistics

5. Zhang, T., Kishore, V., Wu, F., Weinberger, K. Q., & Artzi, Y. (2019). Bertscore: Evaluating text generation with bert. *arXiv, 1904.09675*.

6. Yuan, W., Neubig, G., & Liu, P. (2021). Bartscore: Evaluating generated text as text generation. *Advances in Neural Information Processing Systems, 34*, 27263-27277.

7. NHS Digital (2024). SNOMED CT. <https://digital.nhs.uk/services/terminology-and-classifications/snomed-ct>. Accessed 20 October 2025.

8. Radford, A., Kim, J. W., Xu, T., Brockman, G., McLeavey, C., & Sutskever, I. Robust speech recognition via large-scale weak supervision. In *International Conference on Machine Learning, 2023* (pp. 28492-28518): PMLR
